# Learning semantic categories of L2 verbs: The case of cutting and breaking verbs

**DOI:** 10.1371/journal.pone.0296628

**Published:** 2024-01-19

**Authors:** Noburo Saji, Chunzi Hong, Chong Wang

**Affiliations:** 1 Waseda University, Tokorozawa, Japan; 2 Dalian University of Technology, Dalian, China; Educational Testing Service (ETS), UNITED STATES

## Abstract

To attain native-like proficiency in second-language word usage, learners have to discover intricate semantic categories in the target language. We investigated the factors influencing the development of two aspects of second-language learners’ semantic categories: the category center and category boundary of word meanings. In the experiment, second-language learners of Japanese, whose first language is Mandarin, were asked to produce the best verb for 28 videos depicting various cutting and breaking events. Descriptive analyses were conducted to compare the verb patterns used by second-language learners with those of native speakers. The second-language learners’ verb use pattern suggested their struggle in delineating the semantic ranges of breaking verbs in a native-like manner. Model analyses further revealed that different factors contribute to learning two different aspects of word meanings. The learning category center of word meaning depended on the similarity between the lexical domains in the first and second languages. On the contrary, the success of learning the semantic boundaries of verbs required a large input frequency and smaller semantic coverage, and smaller category ambiguity. The results suggest that constructing a semantic domain in the second language should be evaluated from at least two different aspects of semantic representation.

## Introduction

Cross-linguistic research has long pursued the cross-linguistically shared and language-specific patterns of semantic categorization in various semantic domains, which include the terms for container labels [1], emotion words [2], color words [3], and verbs for locomotion [4]. As languages vary in both the number of semantic categories and the semantic boundaries between them, learners must discover complex semantic structures in the target language. In the last decade, there has been a significant increase in the number of studies exploring the semantic categorization process in the second language (L2). These studies explore how learners learn the semantic relationships between neighboring words, how the relationships differ from those of native speakers, and what drives those differences. The semantic categorization process involves categorization as a domain-general cognitive ability that can be generalized across different parts of speech (i.e., prepositions and nouns) or different target semantic domains (i.e., objects or events) [1,4–7].

The present study aimed to investigate how L2 learners construct semantic categories in L2, taking L2 learners of Japanese whose first language (L1) is Mandarin as a test case. Specifically, we examined what factors contribute to learning how to map the category center of word meanings (*center mapping*) and delineating the category boundaries among synonyms (*boundary delineation*). We specified the four indexes that reflect the quantitative and qualitative characteristics of linguistic input as independent variables and examined whether a different set of factors contributes to the center mapping and boundary delineation processes.

### Two aspects of learning semantic structures in the L2 lexicon

It is widely accepted that L2 learners possess different levels of lexical knowledge [7–11]. One of the most widely accepted divisions is the differentiation between the stage where L2 learners understand the typical meaning of a word (center mapping) and the stage where they understand the semantic boundaries among words (boundary delineation). The term “typical meaning” here is based on the prototype theory by Rosch [12]; the category members have graded membership, and some members are considered more central or typical than others. For example, at one level, an L2 learner of English may possess an understanding that *cut* refers to an event involving the separation of something from its main part. Although this is indeed a typical meaning of *cut*, at this level of knowledge, the learner may inappropriately use this word to describe the action of breaking a rope because they lack knowledge of the semantic boundary between *cut* and *break*. Thus L2 learners must have the appropriate semantic boundaries among already-known words because of the graded degree of belonging to a semantic category, where some members represent typical meanings of the category and others are peripheral and remain ambiguous with the members of neighboring categories.

Nation [11] theorized that the word learning process consists of several interlocking stages, including learning individual items and systems of knowledge. As for the domain of lexical meaning, Nation’s [11] model separated the process of “what items can the concept refer to” from that of “what other word could be used instead of this one” as a different aspect of lexical acquisition [11]. Henriksen [10] also conceptually separated the processes of lexical learning in L2 into “form-meaning mapping” and “network building.” This model predicts that L2 learners generally identify the connection between the form and the meaning in the L2 lexicon and then establish the semantic boundaries between the form–meaning pairs [13]. The authors also point out that studies on word learning have to distinguish between achieving the typical meaning of a word (center mapping) and identifying semantic relationships between neighboring categories (boundary delineation) as separate processes [14–21].

Many studies have addressed how learners achieve center mapping and boundary delineation. In particular, the center mapping process has long attracted the interest of researchers. L2 learners generally acquire the typical meanings of words earlier than peripheral ones, as the typical meaning of a word is more perceptually salient or receives a higher frequency of input than the atypical meaning [17,22,23]. Ijaz [17], for example, demonstrated that adult speakers of German who learned English spatial prepositions more closely approximated native speakers of English in typical meanings of “on” and “over” than in atypical ones. Recent studies, in contrast, have emphasized the difficulty of acquiring native-like semantic boundaries. Learners’ understanding of L2 words is imprecise, and they frequently over- or under-extend L2 word meanings [16,17,20,21,24]. Saji and Imai [7] examined how L2 Mandarin learners whose L1 was Japanese or Korean understood the meanings of Mandarin verbs for carrying and holding. In the experiment, participants watched videos of 13 carrying/holding events and were required to produce the verb they thought most appropriately applied to each event. The results showed that L2 learners could apply the appropriate verbs for typical actions, which native speakers accepted as the best referent for the verb. However, they failed to produce the appropriate verbs for the peripheral events for which native speakers considered two or more verbs acceptable referents.

### Factors contributing to the process of constructing lexical structures in L2

Previous studies have proposed possible factors that may influence the process of learning L2 words concerning the center mapping and boundary delineation processes. Some of these variables are specific to L2 lexical learning, while others are general to category learning. In the following, the candidate factors that might contribute to the process of semantic categorization are listed: 1) *input frequency* (the token frequency of each word to which the L2 learners are exposed), 2) *L1–L2 semantic similarity* (the degree to which the lexical structure of L1 is similar to that of L2), 3) *category size* (the range of the referents named by each word), and 4) *category ambiguity* (the degree of disagreement in naming each word across speakers). These factors can be broadly divided into two categories: quantity factors of input (1) and quality factors of input (2–4).

First, input frequency has long been considered a significant factor in both L1 and L2 vocabulary acquisition [14,15,24]. High-frequency words are easier to acquire and retain than low-frequency words. Saji et al. [15] demonstrated that input frequency has a clear effect on the center mapping process. However, others have reported that the frequency effects cannot be considered in isolation. Gass and Mackey [25] argued that factors such as perceptual saliency, semantic complexity, and semantic similarities between L1 and L2 may interact with frequency and contribute to the ease of learning.

The quality of the linguistic input may also matter as much as, or even more than, the quantity of input in lexical learning. One factor that has been intensively studied is the effects of L1 transfer, where the degree of similarity between the semantic structures of L1 and L2 determines the ease of learning a target language (L1–L2 semantic similarity) [17,19,24,26,27]. L2 learners often rely on their L1 knowledge, whether consciously or unconsciously, to establish word meanings, particularly when the semantic structures in L1 are different from those in L2 [1,16,17,19,26]. In her Modified Hierarchical Model, Pavlenko [28] proposed that learners’ conceptual categories can be classified into two categories: cross-linguistically shared categories and language-specific categories (i.e., L1-specific categories and L2-specific categories). The model hypothesizes that if a word in L1 and its counterpart in L2 partially share conceptual categories (i.e., the meaning of L2 words consists of both cross-linguistically shared and L2-specific concepts), learners fail to restructure the concepts of the L2 word meaning because the word meaning in L2 is understood through L1-specific knowledge [20].

Furthermore, research on category learning from a domain-general perspective suggests that category size and category ambiguity may affect learning semantic structures. As for category size, prototypes are learned more easily with categories of large size than with those of small size [29–31]. Specifically, the diversity of exemplars in large categories facilitates accessibility to prototype members because the greater the number and variety of stored exemplars, the higher the likelihood that they share features [32–34]. This finding applies to L2 learning, as L2 learners readily use words with general meanings that can have a broad range of references [17,35]. As for category ambiguity, when a word has many neighboring words with partially overlapping meanings, the same referent is likely to be labeled with different names, leading to ambiguous semantic boundaries [14,15,20,36,37]. For example, the referent of the word “black” in English (i.e., black color) is rarely labeled by other color words, but the referent of “orange” can be labeled as “yellow,” “brown,” “tangerine,” “amber,” and so on. Therefore, it can be quite difficult for learners to understand the appropriate boundaries of meanings of each word. Category ambiguity affects learning ease, as shown in L1 lexical acquisition studies [1,14,15], but its impact on L2 lexical learning is not well understood.

Although previous studies showed evidence for possible factors, these factors were separately evaluated. Since these factors are concurrently at work in the real learning situations, they must be examined simultaneously.

### Semantics in cutting/breaking events

The domain of cutting/breaking events offers an interesting test case for examining the construction of semantic categories in L2 because previous cross-linguistic studies have revealed that the domain includes both cross-linguistically shared and language-specific aspects [5,38]. Majid et al. [38] conducted a comprehensive survey to investigate the cross-linguistic commonalities in the categorization of cutting and breaking events in 28 diverse languages. Participants watched 61 video clips that depicted a broad range of cutting and breaking events and were asked to label the event in each video. The analyses revealed that in all languages, events in which the locus of separation was highly predictable were distinguished from those in which it was not; for example, the names for events of breaking a plate with a hammer were distinct from those for other cutting/breaking events. However, language-specific semantic boundaries were observed in the number of categories and the placement of their boundaries. Wang et al. [39] also investigated cross-linguistically shared and language-specific aspects of the meanings of cutting and breaking verbs in Mandarin and Japanese. In the experiment, native speakers of Japanese and Mandarin were asked to produce the best verb for 28 videos depicting various cutting and breaking events. Consistent with Majid et al. [38], the results showed that the two languages commonly distinguished the cutting events (the locus of separation was highly predictable) from the breaking ones (the locus of separation was not predictable) by different verbs. As for language specificity, Mandarin participants finely differentiated several cutting events with different verbs based on the features of “instruments for cutting.” For example, Chinese speakers produced *qie1* for the events of cutting with a single-blade instrument, *ju4* for the events of cutting with a saw, and *jian3* for the events of cutting with a two-bladed instrument. Japanese participants labeled these events with a single verb, *kiru* (“cutting an object with edged tools”). In contrast, Japanese participants differentiated the manual breaking events more finely than did Mandarin-speaking participants, using verbs such as *yabuku* (“tearing a thin object by hand”), *chigiru* (“tearing an object into small pieces by hand”), and *saku* (“ripping up an object by hand”). Malt and Sloman [11] referred to such cases as *cross-cutting categories* and suggested that the cultural or linguistic idiosyncrasies of the categories would impede category learning [16,28]. Mandarin-speaking learners of Japanese are a good test case because their learning process includes cross-cutting categories: both simple L1 system to the complex L2 system and complex L1 system to the simple L2 system.

### The present study

The present study aims to investigate which factors influence the process of constructing semantic representations in L2 from two aspects: center mapping and boundary delineation. The analyses were conducted in two steps. First, we examined how the patterns of verb use by native speakers differed from those of L2 learners (Analysis 1). In line with previous research [11,16,21,28], we used multivariate analyses to visualize the verb use patterns of L2 learners and clarify similarities or differences with those of native speakers. Next, we examined which factors contributed to L2 learners’ verb use in terms of center mapping and boundary delineation (Analysis 2). Here, our approach is unique in that we simultaneously assessed the quantitative and qualitative aspects of linguistic input (but see Zinszer et al.’s study [40], which aimed to disentangle the effects of several factors concerning individual language history in predicting the nativelikeness of L2 lexical semantics).

Based on previous studies, we established three hypotheses regarding the effects of the factors. First, input frequency and category size forecast the ease of center mapping [24,25,39,41]; words that are input with high frequency and have large category sizes are easy to map to their typical reference. Second, category ambiguity predicts the process of boundary delineation; when L2 words have ambiguous boundaries with neighboring words, delineating appropriate boundaries becomes difficult [7,15]. Third, as the cutting/breaking verbs in Mandarin and Japanese share cross-cutting categories, L1–L2 semantic similarity impedes the lexical learning of L2 in both center mapping and boundary delineation [20,28].

We recruited L2 learners from a university in China, as the process of semantic reorganization is particularly challenging for learners who study L2 in an L1-speaking environment [20,40]. In the experiment, we presented videos of cutting and breaking events to participants and asked them to produce the most suitable verb. The materials and procedure were borrowed from Wang et al. [39], who investigated the cross-linguistic differences and commonalities between Japanese and Mandarin in the semantic system of cutting/breaking verbs. We calculated the scores of center mapping and boundary delineation from the production data by L2 learners of Japanese and fed them into the model analyses. As for the independent variables, we used the production data from native speakers of Japanese and Mandarin collected by Wang et al. [39]. We used the data to calculate the scores of category size, category ambiguity, and L1–L2 similarity. Input frequency was collected from the textbooks the learners used.

## Methods

### Participants

The present study included 29 native Mandarin Chinese speakers (female = 8; average age 21.8 years) who were undergraduate and graduate students at the Dalian University of Technology, as well as 21 native Japanese speakers (female = 12; average age 21.9 years) who were undergraduate students at Hirosaki University. All participants were functional monolinguals in their daily lives and had no experience studying Japanese/Mandarin. The data were taken from Wang et al. [39].

The L2 learner participants consisted of 30 lower-proficiency (female = 12) and 30 higher-proficiency (female = 14) Mandarin-speaking learners of Japanese, who were newly recruited for this study from the Japanese language department at the Dalian University of Technology. None of them had experience of residing in Japan (Table 1). The high-/low-proficiency classification was based on their scores on the Simple Proficiency-Oriented Test (SPOT) Version 2 for intermediate–advanced learners [42], which measures proficiency in Japanese. The validity and reliability of the SPOT have been confirmed [42,43]. For instance, studies have revealed that the SPOT score strongly correlates with the American Council on the Teaching of Foreign Languages-Oral Proficiency Interview (ACTFL-OPI) class (*r* =. 81 [44]; *r* = .73 [45]). The average of the SPOT scores of higher-proficiency participants (*M* = 48.3, *SD* = 5.41, range: 42–61) was significantly higher than that of lower-proficiency participants (*M* = 26.6, *SD* = 4.72, range: 13–33) (*t* = 16.56, *df* = 58, *p* < .001, d = 4.2). According to Suzuki [45], who constructed the regression model to predict the ACTFL-OPI class from the SPOT score, the lower- and higher-proficiency learners in the current study are classified as “novice” and “intermediate” ACTFL-OPI classes, respectively.

**Table 1:** Basic information about the L2 learner participants (N = 60).

Proficiency groups	Length of studying Japanese			SPOT score (max 65)		
	M	Range	SD	M	Range	SD
High (n = 30)	3.27	2.50–5.50	0.9	48.3	42.00–61.00	5.42
Low (n = 30)	2.5	2.50–2.50	0	26.57	13.00–33.00	4.72

### Materials

**Fig 1:**
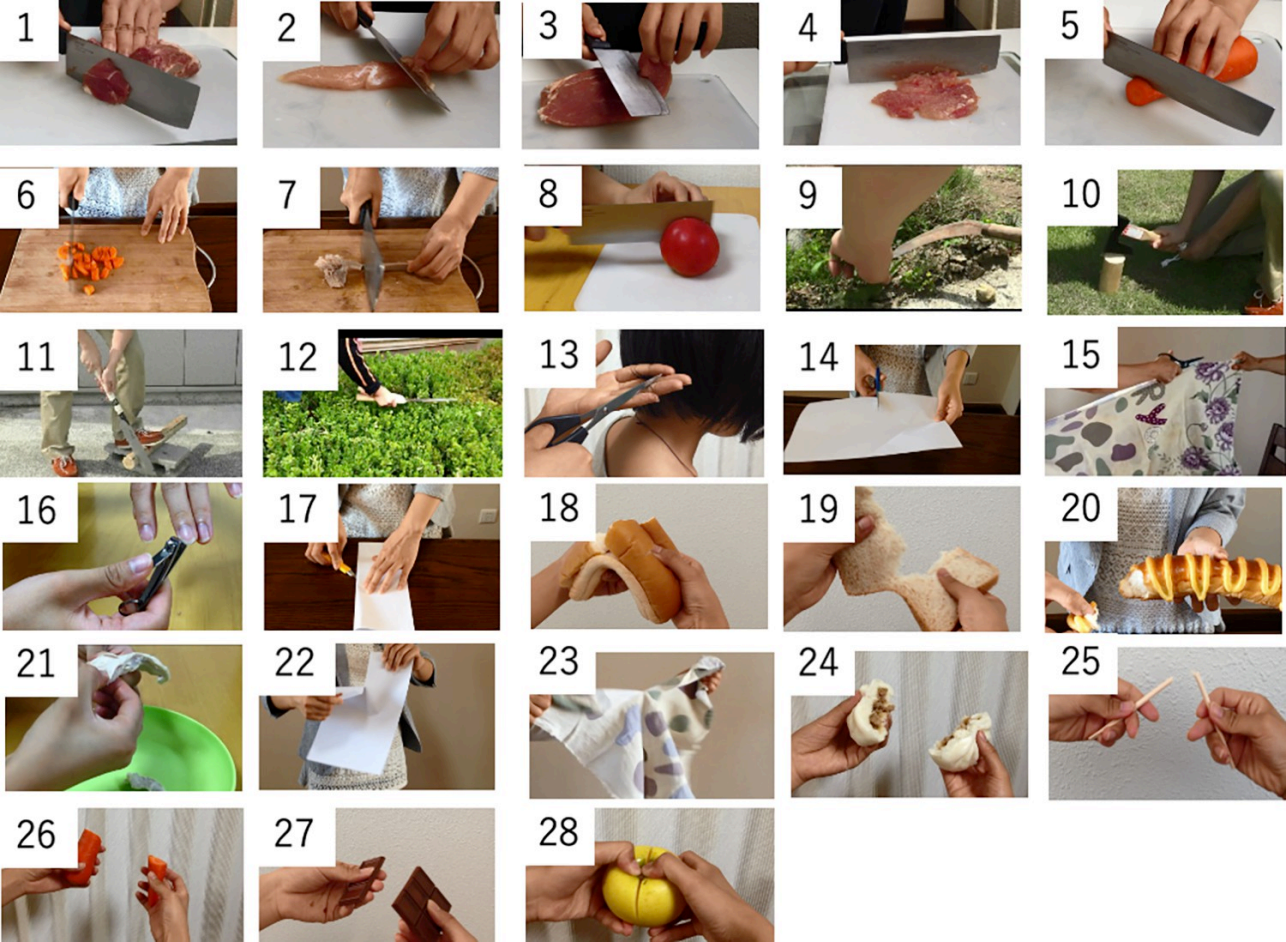
Stimuli videos.

The stimuli videos were borrowed from Wang et al. [39]. Here, a set of 28 videos of cutting and breaking events were selected based on the findings of Majid et al. [38] and Chen [46], as the videos correctly depict the events described by Chinese/Japanese verbs. The cutting and breaking events varied across five parameters: “predictability,” “direction,” “dimension of the objects,” “rigidness of the objects,” and “result state of the objects” (Table 2; see Fig 1 for sample video snapshots). “Predictability” differentiates events in which the locus of separation is predictable from events in which it is not. “Direction” means the direction of the separation: that is, crosswise vs. in the other direction. The parameters of the objects were rigidness, dimension, and result state. Rigidness represents whether the object is rigid or flexible. According to dimension, objects were categorized into three types: one-dimensional (e.g., rope), two-dimensional (e.g., cloth), and three-dimensional (e.g., apple). Result state represents the number of pieces into which the objects were broken in the events [38,46]. Finally, we performed a video check with the Mandarin and Japanese native-speaker groups to confirm that the video content consisted of daily activities that all L2 learners could easily understand.

**Table 2:** List of stimuli videos.

ID	Events	Predictability	Direction	Dimension of the object	Rigidness of the object	Resulting states of the objects
1	Cutting meat with a kitchen knife	predictable	vertical	1	flexible	two pieces
2	Slicing meat with a kitchen knife	predictable	horizontal	1	flexible	two pieces
3	Chopping a bit of meat with a kitchen knife	predictable	horizontal	1	flexible	two pieces
4	Chopping meat with a kitchen knife	predictable	vertical	1	flexible	more than two pieces
5	Slicing a carrot with a kitchen knife	predictable	vertical	1	rigid	two pieces
6	Chopping a carrot with a kitchen knife	Predictable	vertical	1	rigid	more than two pieces
7	Chopping a bone with a Mandarin chef’s knife	not predictable	vertical	1	rigid	two pieces
8	Cutting a tomato with a kitchen knife	predictable	vertical	3	flexible	two pieces
9	Cutting grass with a sickle	predictable	horizontal	1	flexible	two pieces
10	Chopping a log with an ax	not predictable	vertical	1	rigid	two pieces
11	Sawing a log	predictable	vertical	1	rigid	two pieces
12	Cutting grass with shears	predictable	horizontal	1	flexible	more than two pieces
13	Cutting hair with scissors	predictable	horizontal	1	flexible	two pieces
14	Cutting a piece of paper with scissors	predictable	vertical	2	flexible	two pieces
15	Cutting a piece of cloth with scissors	predictable	horizontal	2	flexible	two pieces
16	Clipping nails with nail clippers	predictable	vertical	2	rigid	two pieces
17	Cutting a piece of paper with a box cutter	predictable	horizontal	2	flexible	two pieces
18	Tearing a loaf of bread in half by hand	not predictable	vertical	1	flexible	two pieces
19	Tearing a slice of bread by hand	not predictable	horizontal	2	flexible	two pieces
20	Tearing a piece out of a baguette/roll by hand	not predictable	horizontal	2	flexible	two pieces
21	Tearing chicken by hand	not predictable	horizontal	2	flexible	two pieces
22	Tearing a sheet of paper in half	not predictable	horizontal	2	flexible	two pieces
23	Tearing a piece of cloth by hand	not predictable	horizontal	2	flexible	two pieces
24	Tearing/dividing a steamed meat bun by hand	not predictable	vertical	3	flexible	two pieces
25	Breaking/snapping a chopstick by hand	not predictable	vertical	1	rigid	two pieces
26	Breaking/snapping a carrot by hand	not predictable	vertical	1	rigid	two pieces
27	Snapping a bar of chocolate by hand	not predictable	vertical	2	rigid	two pieces
28	Splitting an apple by hand	not predictable	vertical	3	rigid	two pieces

### Procedure

All procedures used in the current experiment were approved by the Ethical Committee of Kamakura Women’s University (#14028). Verbal consent was obtained from all participants before the experiment. Each participant was individually assessed in a quiet room at their respective universities by an experimenter who spoke Mandarin or Japanese as their L1. The 28 stimuli videos were presented on a computer screen in random order. Instructions were provided in the participant’s native language and shown on the computer monitor. The native speakers of Japanese and Mandarin who were not Japanese learners were asked to orally describe the event in the videos in their native language (Japanese or Mandarin). The Japanese learners were also asked to describe what was occurring in the videos in Japanese. In the coding process, one verb denoting the cutting/breaking event (the principal action) in the video was extracted for each participant. For example, for a participant’s response of “*hamono* (edged tool) *wo* (particle) *motte* (have) *ninjin* (carrot) *wo* (particle) *kiru* (cut),” we included only the verb *kiru* (“cutting an object with edged tools”) for analyses. Intercoder reliability based on agreement percentage was 97%, indicating a high level of agreement between the two coders. Disagreements on coding were resolved through discussion and by consulting other linguists and, on several occasions, by consulting directly with the participants.

## Results

In Analysis 1, we examined how naming patterns differed between Mandarin and Japanese, and between native speakers and L2 learners. For this purpose, we utilized the multidimensional scaling (MDS) solution to visualize verb usage patterns and discuss what kind of semantic dimensions native speakers and L2 learners adopt when using verbs. In Analysis 2, we performed model analyses to determine what factors contribute to center mapping and boundary delineation. The model examined the impact of category size, category ambiguity, and L1–L2 similarity, calculated from the production data of the native speakers, and input frequency was collected from the textbooks used in the classroom.

### Analysis 1: Naming pattern of verb use by native speakers and L2 learners

Before presenting the naming pattern by native speakers and L2 learners, we report the descriptive statistics on the number of verb types produced by participants. For the 28 stimuli videos, the averages of verb types produced by each participant were calcurated. In counting verb types, if a participant produced *kiru* (20 times), *chigiru* (four times), *yabuku* (two times), and *oru* (two times) to denote a total of 28 events, the number of verb types they produced was counted as four. The averages of verb types were 10.24 and 8.52 for the native speakers of Mandarin and Japanese, respectively, and 6.77 and 4.7 for the high- and low-proficiency learners.The one-way ANOVA revealed a significant effect of four participants’ backgrounds on the averages of verb types (*F*(3, 106) = 34.41, *p* < .00, η2 = .49). A post-hoc test revealed that Mandarin speakers produced significantly more verb types than did Japanese speakers (Bonferroni corrected: *t* = -4.3, *df* = 48, *p* < .05, d = 1.24). This suggests that the native speakers of Mandarin delineated the 28 events more precisely than did the native speakers of Japanese. The averages significantly differed between the higher- and lower-proficiency learners (Bonferroni corrected: *t* = 3.0, *df* = 58, *p* < .05, d = 0.78), but both averages were significantly lower than those of native speakers of Japanese (Bonferroni corrected: *t* = -2.72, *df* = 49, *p* < .01, d = .77 for higher-proficiency learners; *t* = -.5.65, *df* = 49, *p* < .01, d = 1.61 for lower-proficiency learners). The L2 learners did not produce as many verbs as did the native speakers, although the higher-proficiency learners had a larger vocabulary than did the lower-proficiency learners.

Next, to visualize the pattern of semantic categorization with the verbs produced by participants, we adopted the MDS solution [47]. MDS provides a geometrical representation of patterns of similarity on dimensions that are extracted to maximize goodness of fit such that inter-point distances on the multidimensional space correspond to dissimilarities between objects. For the analyses, we first created a similarity matrix for the four groups of participants (i.e., native speakers of Japanese, native speakers of Mandarin, higher-proficiency learners of Japanese, and lower-proficiency learners of Japanese). Each matrix comprised 28 rows and 28 columns, each representing the stimulus events. In addition, each cell contained the number of times the two events were named with the same verb. In this study, MDS visually demonstrates how speakers of each language categorize the 28 cutting and breaking events with verbs.

**Fig 2:**
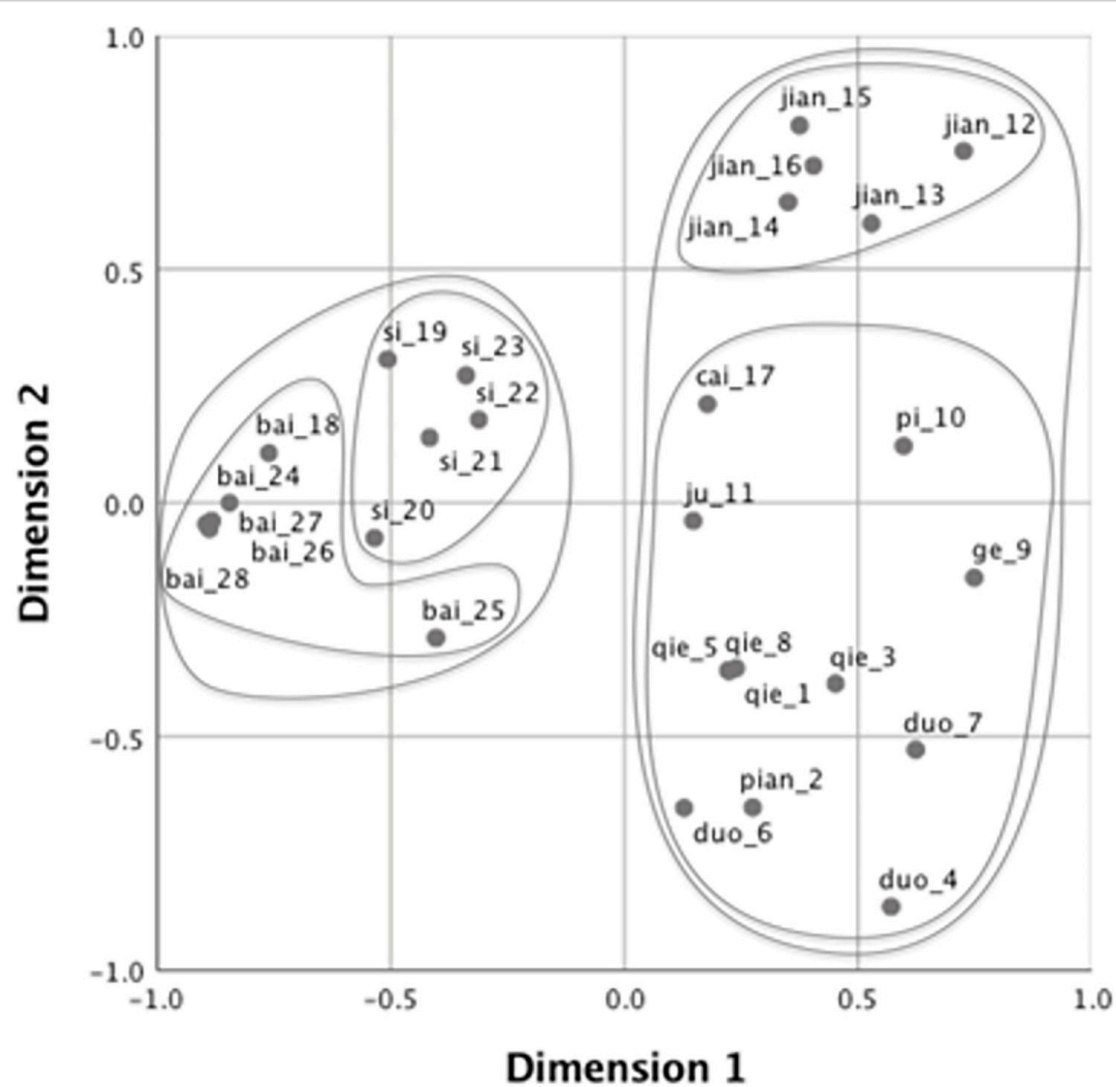
Multidimensional scaling solution for native speakers of Mandarin.

**Fig 3:**
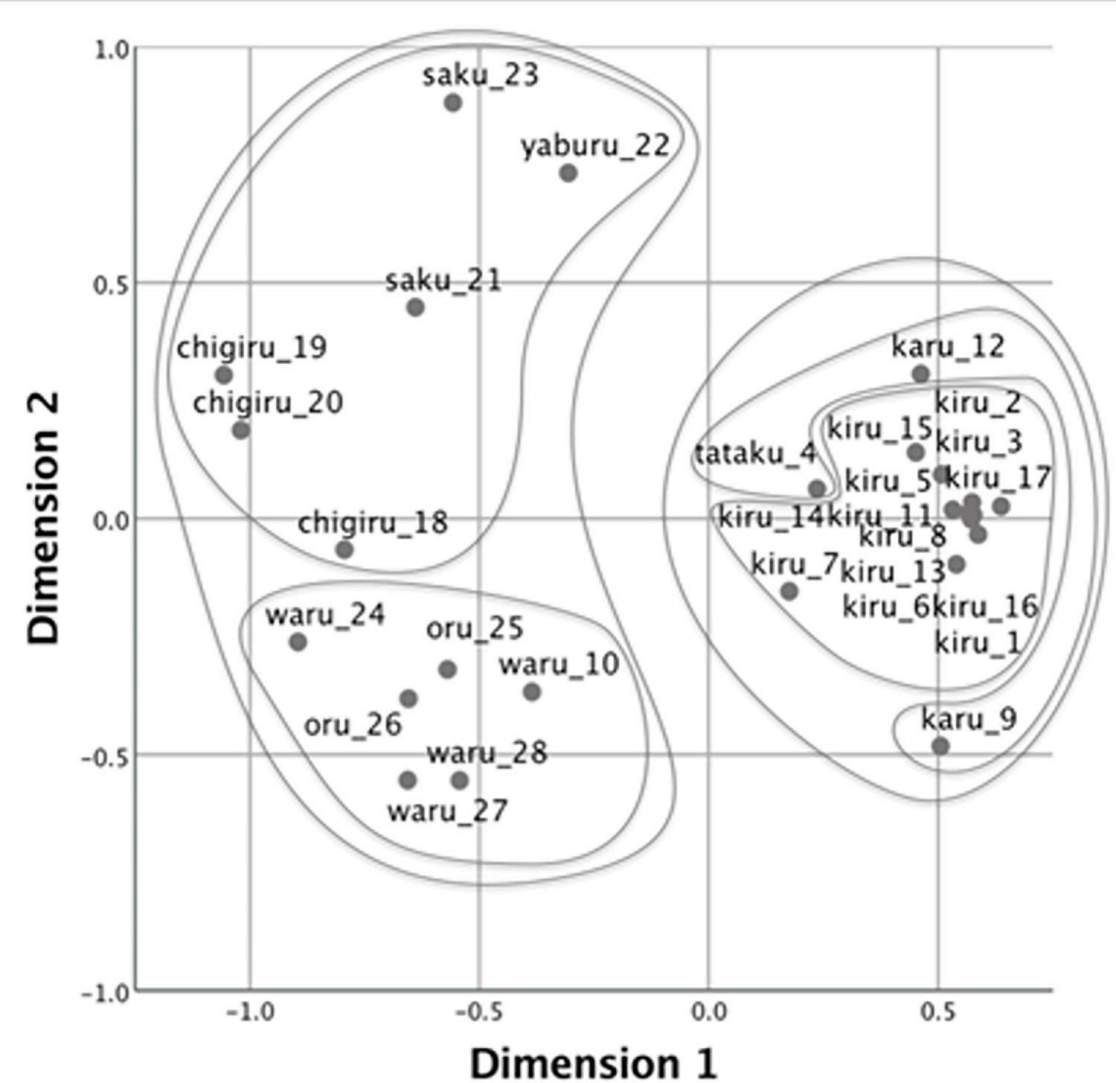
Multidimensional scaling solution for native speakers of Japanese.

**Fig 4:**
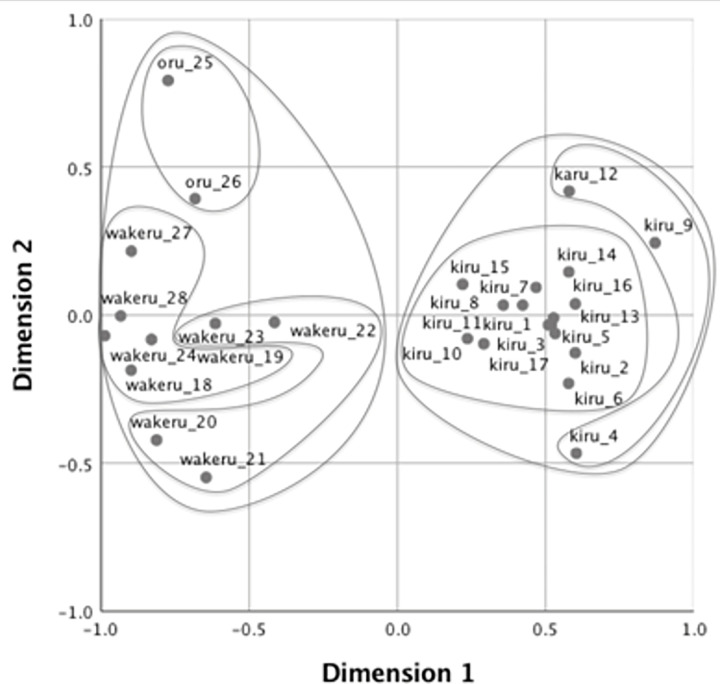
Multidimensional scaling solution for high-proficiency learners.

**Fig 5:**
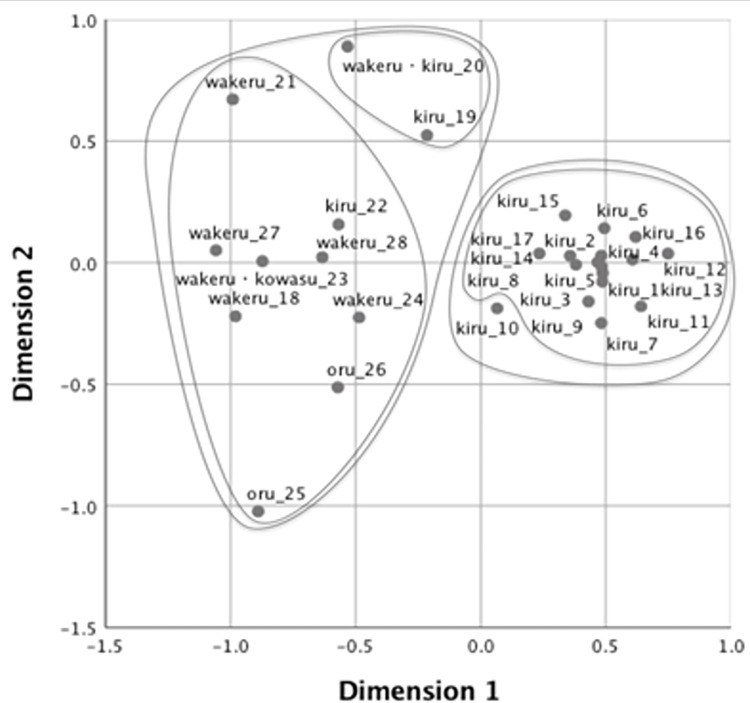
Multidimensional scaling solution for low-proficiency learners.

The stress value represents how well a set of data is represented by the predicted values by the MDS model. Kruskal [48] suggested that the goodness of fit is sufficient if the value is under 0.1. We employed a two-dimensional solution for all groups, as the stress value was acceptable (.08 for native speakers of Japanese, .07 for native speakers of Mandarin, .07 for higher-proficiency learners, and .08 for lower-proficiency learners. Figs 2–5 show the MDS configurations for the four groups. Each point on the configuration represents one of the 28 events, and the distance between any two points represents the similarity between them. Two events are closely located if many participants applied the same verb, and far apart if few participants used the same verb. The points were labeled with the same dominant (most frequently produced) verbs and video IDs (see S1 Table for the list of dominant verbs for the 28 videos). Furthermore, additive tree clusters were drawn on each from the MDS results [49,50]. Only the top two levels of clusters are provided for ease of viewing.

We initially examind the categorization pattern of native Mandarin and Japanese speakers in their respective languages. Figs 2 and 3 demonstrate the results concerning native speakers of Mandarin and Japanese, respectively. Consistent with Wang et al. [39], the speakers of the two languages distinctly differentiated the cutting events performed with instruments (Videos 1 to 17) from those involving breaking by hand (Videos 18 to 28) in the primary dimension, except Video 10 (chopping a log with an ax), which was positioned on the left side in Japanese. Language specificity was observed in the secondary dimensions, which further differentiated among the events that were already distinguished along Dimension 1. In Mandarin, the secondary dimension primarily reflected the types of edged tools used. Cutting events with scissors were grouped (Videos 12–15) in the positive direction as *jian3* (剪: “cutting with two-blade instruments”), while the events with a knife (Videos 1–8) were clustered in the negative direction as *qie1* (切: “cutting with a knife”), *duo4* (剁: “chopping with a knife”), or *pian4* (片: “slicing with a knife”). Conversely, in Japanese, Dimension 2 likely reflected the rigidity of the objects, with cutting and breaking events for rigid objects (Videos 25 to 28) named *waru* (割る: “splitting a two- or three-dimensional object into several pieces”) and *oru* (折る: “snapping a one-dimensional object”) in the negative direction, and events for flexible objects (Videos 18, 21, 23) were named *yaburu* (破る: “tearing a thin object by hand”) and *saku* (裂く: “ripping up an object by hand”) in the positive direction.

Figs 4 and 5 present the naming patterns of L2 learners for the high- and low-proficiency groups, respectively. In Dimension1, L2 learners were able to label the cutting and breaking events with the edged tool as *kiru* (切る: “cutting an object with an edged tool”) and the events by hand as *wakeru* (分ける: “dividing objects into several pieces”) and *oru* (折る: “snapping a one-dimensional object”). The videos categorized by dimensions are the same as those used by native speakers. However, the naming patterns of both learner groups in Dimension 2 were different from those of native speakers. In both Figs 4 and 5, Video 25, named *oru* (折る: “snapping a one-dimensional object”), and Videos 20 and 21, named *wakeru* (分ける: “dividing an object into several pieces”), were positioned on the edges of Dimension 2, indicating that these events were first differentiated from other events of cutting and breaking by hand. Higher-proficiency learners were more likely to begin creating native-like semantic categories. They closely grouped Videos 18–20, 24, 27, and 28, which native speakers of Japanese also grouped as *chigiru* (ちぎる: “tearing an object into small pieces by hand”) and *waru* (割る: “splitting a two- or three-dimensional object into several pieces”), although the events were equally spaced along the secondary dimension in the lower-proficiency learners’ graph.

It is noteworthy that L2 learners frequently used *wakeru* (分ける: “dividing an object into several pieces”), though native speakers did not use the verb. In previous research, it has been documented that when learners do not have a precise vocabulary to describe a referent, they may use a general-purpose verb that is not used by native speakers [7,51–53]. The meaning of *wakeru* in Japanese is a more general-purpose verb than the other cutting/breaking verbs, as the verb does not conflate the manner of dividing or type of tools used in its meaning. Participants of the study might have used *wakeru* because they did not know the specific verbs as *saku* or *waru*, but at the same time knew that it was not the event of *kiru* because the actor does not use edged tools.

Taken together, the MDS solution showed that L2 learners could readily identify the most general boundary in L2 verbs (i.e., the events by edged tool and those by hand). However, even higher-proficiency learners still struggled to delineate the boundaries within the more specific events as cutting/breaking events by hand. In Analysis 2, we aim to explore why some verbs are easier to learn than others and what semantic properties of verbs contribute to the ease of learning L2 verbs.

### Analysis 2: What factors predict the ease of learning L2 verbs in constructing lexical representation?

#### Independent variables

The goal of the analysis was to examine what factors contribute to the ease/difficulty of L2 verb learning. Here, we assumed that the four factors presented in the Introduction contribute to the learning process: the frequency of verb input (input frequency), the range to which the verb is applied (category size), the degree of boundary overlap with neighboring words (category ambiguity), and the degree of similarity between L1 and L2 verbs (L1–L2 semantic similarity).

**Fig 6:**
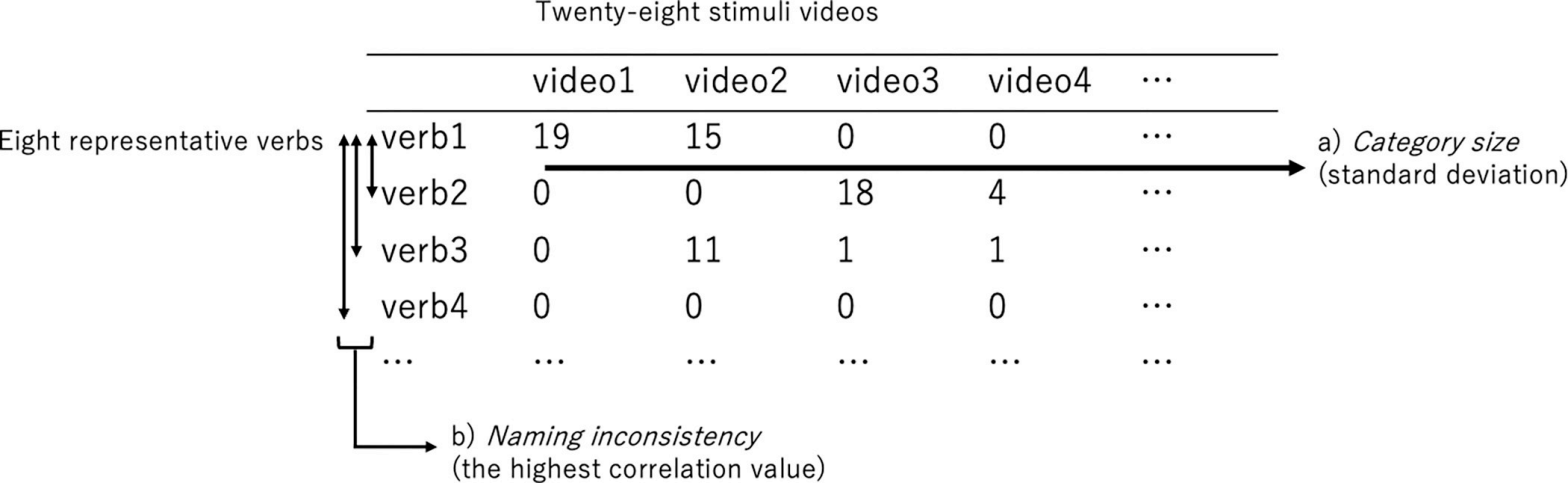
Calculation Process for the scores of category size and category ambiguity.

We quantified the four factors for each of the eight representative verbs that were dominantly produced for the 28 videos by native speakers of Japanese: *kiru*, *chigiru*, *waru*, *yaburu*, *saku*, *oru*, *tataku*, and *karu* (see Appendix 1 for the dominantly produced verbs for the 28 videos). We estimated the input frequency of each of the eight cutting and breaking verbs by counting the token frequency in the five textbooks the learners used in the classroom (*Basic Japanese 1–4*:基礎日本語1–4). Category size and category ambiguity were computed using the production data of native speakers of Japanese. First, to quantify the indices, an 8 x 28 matrix was created to represent the frequency of eight verbs produced for the 28 videos, using the production data by the native speakers of Japanese (see Fig 6 for the calculation process for the two variables). Second, for the calculation of category size, we used vector (a) in Fig 6, which represents the number of verbs produced in the 28 videos. If a verb is concentrated on a particular event, the deviation is high. In contrast, if a verb is equally produced in several events, the deviation is low. For example, *kiru* (切る: “cutting an object with an edged tool”) was produced for 17 events, while *karu* (刈る: “mowing grass”) was produced for two events. In this case, the higher score (9.24) was assigned for *kiru* than for *karu* (3.21). Third, we attempted to quantify whether a verb has a counterpart that carries a similar meaning by category ambiguity. In the case of synonyms, learners could confuse the boundaries between two verbs. For this purpose, we first calculated correlation values for all possible pairs of the eight-row vectors (b) in Fig 6. The correlation values reflect the similarity between two verbs in terms of the naming patterns for the events. Then, we identified the verbs that produced the highest correlation values with each of the eight representative verbs and indexed these eight correlation values as category ambiguity. For example, the verb that obtained the highest correlation value with *saku* (裂く: “ripping up an object by hand”) was *yaburu* (破る: “tearing a thin object by hand”), and the correlation was .34. On the contrary, *kiru* (切る: “cutting an object with an edged tool”) was the most correlated with *tataku* (叩く: “chopping an object into fine pieces”), but the correlation was substantially low (-.05). In this case, *saku* had the more ambiguous counterparts (i.e., *yaburu*) than *kiru* (i.e., *tataku*). Fourth, L1–L2 semantic similarity was calculated by a similar idea as with category ambiguity described above. Here, we calculated correlations for all pairs of eight Japanese verbs and 10 representative Chinese verbs, and then adopted the maximum correlation values with eight Japanese verbs as the L1–L2 semantic similarity score; if a verb in Japanese has a very similar counterpart in Chinese, the correlation is high. For example, *karu* (刈る: “mowing grass”) in Japanese has a similar meaning to *ge1* (割:cutting with a single-blade instrument, slowly back and forth) in Chinese, and the correlation was .75. On the contrary, the most similar Chinese verb with *chigiru* in Japanese was *si1* (撕: “pulling a flexible object forcefully in a horizontal direction by hand”), but the correlation was .51. In this case, we can predict that the transfer of L1 knowledge readily occurs in the former case (see Table 3 for the specific numbers of independent variables). The verb *karu* was excluded from the following analyses because the input frequency was 0, and the learners were expected not to know this word (see Table 3).

**Table 3:** Independent variables for eight representative verbs in Japanese.

Verb	Input frequency	Category size	Category ambiguity	L1–L2 semantic similarity
*kiru*	53	9.24	-0.05	0.52 (correlation with *que4*)
*chigiru*	4	4.93	0.09	0.51 (correlation with *si1*)
*yaburu*	14	2.08	0.34	0.64 (correlation with *pi1*)
*waru*	6	6.23	0.13	0.67 (correlation with *si1*)
*karu*	0	3.41	-0.05	0.75 (correlation with *ge1*)
*oru*	4	6.23	0.13	0.53 (correlation with *bai1*)
*tataku*	6	2.08	-0.05	0.74 (correlation with *duo4*)
*saku*	1	3.44	0.34	0.75 (correlation with *si1*)

#### Dependent variables

**Fig 7:**
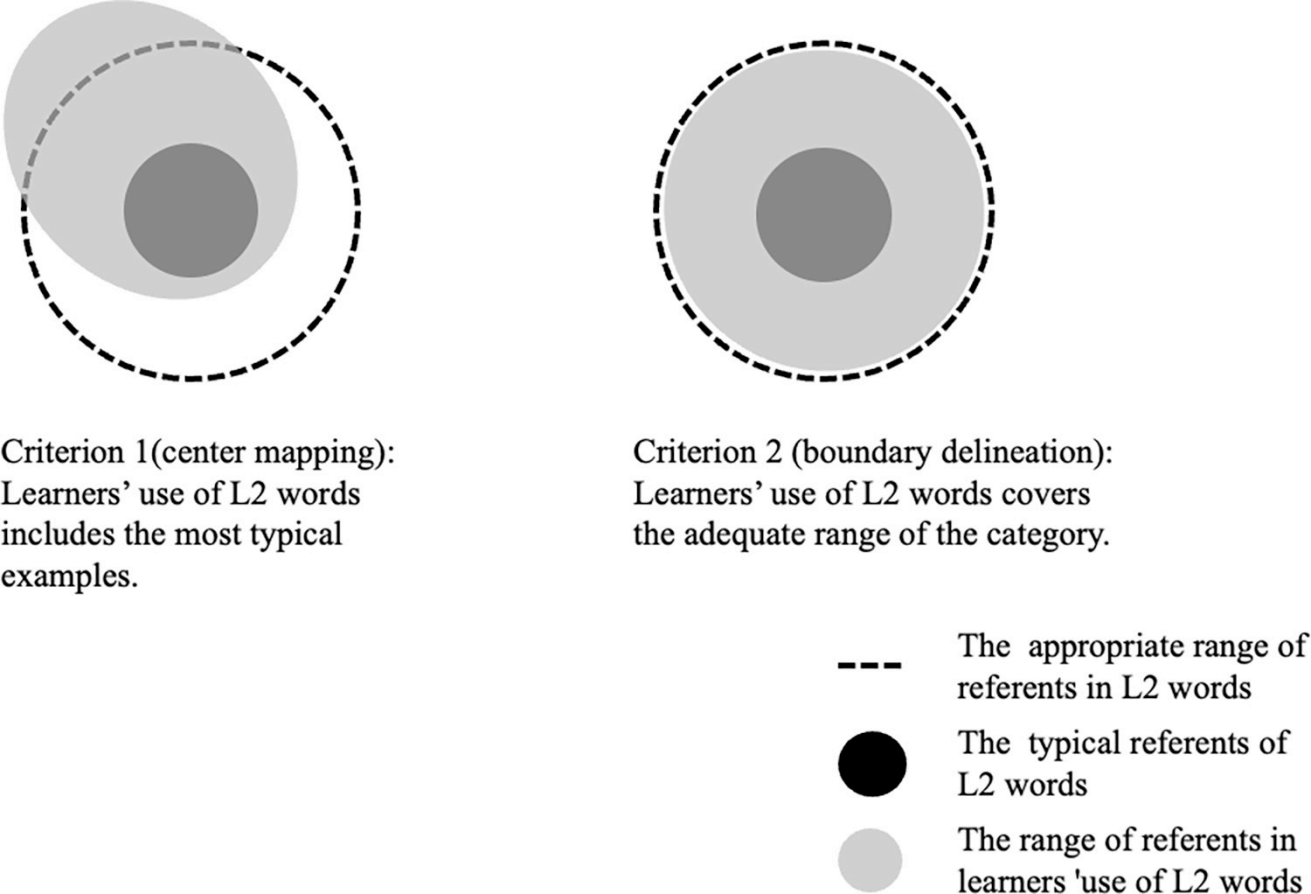
Center mapping and boundary delineation.

We aim to determine whether the four factors contribute to different aspects of semantic representation: center mapping and boundary delineation. First, L2 learners must find the typical references of the target word as shown in Criterion 1 in Fig 7. Here, the referents of the word produced by L2 learners are, at least in part, identical to the typical referents for native speakers, despite the other parts being over/under-extended. To quantify the criterion, we examined whether each L2 learner could apply the eight verbs to the videos that Japanese native speakers accepted as the best referent for the verb, as determined by the highest dominance rate in Appendix. For instance, if an L2 learner produced the verb *oru* (折る: “snapping a one-dimensional object”) for Video 25, which is the best referent for *oru* for native speakers, the response was coded “1;” if not, the response was coded “0.” Concerning boundary delineation, we aimed to check whether the verb used by L2 learners covered the native-like ranges of referents without over/under-extending their meanings as described in Criterion 2 in Fig 7. For this purpose, we adopted the *f-measure*, popularly used in the context of information retrieval to evaluate how well the system can extract relevant instances from the database. In general, the f-measure evaluates the accuracy of binary classification such as correct/incorrect instances. In the current case, we utilized the measure to evaluate the correctness of the participants’ verb choices. The f-measure is defined as the harmonic mean of *precision* and *recall*. In the field of information extraction, precision is defined as the number of true positive results divided by the number of all retrieved references. Recall is defined as the number of positive results divided by the number of all samples that should have been identified as positive. The two measures are basically in a tradeoff relationship; if the precision score increases, the recall score decreases, and vice versa. Thus, the harmonic mean of the two measures is adopted to represent the accuracy of classification [15,54]. In the current case, we first defined the native-like responses for each video, according to the dominant names of the native speakers. Then we computed the f-measure score for eight representative verbs produced by each participant. For example, a participant produced *chigiru* for five videos (ID 18–23), and the dominant names for the five videos by the native speakers were *chigiru* (ID 18), *chigiru* (ID 19), *chigiru* (ID 20), *saku* (ID 21), *yaburu* (ID 22), and *saku* (ID 23). In this case, the precision score for *chigiru* was 3/5 (true positives/retrieved instances), and the recall score was 1 (true positives/all correct instances). The f-measure score assigned to *chigiru* by the participant was the harmonic mean of the two scores (.67). Consequently, the verb use of L2 learners is both not overextended and underextended and the f-measure score is higher. We calculated the f-measure of eight cutting/breaking verbs for every learner when the produced verb included at least one correct response, because if the referents of a verb included no correct responses, the f-measure for the term could not be calculated.

#### Results

The first model examined the factors contributing to center mapping. A logistic mixed-effects model was implemented to predict whether L2 learners could map L2 words to the typical response (i.e., center mapping), specifying input frequency, category size, category ambiguity, and L1–L2 semantic similarity in the model as fixed effects, using the *lme4* package with R [55]. Individual participants were specified as random effects at intercept. Table 4 shows that the effect of L1–L2 semantic similarity was significant. L1–L2 semantic similarity contributed to the model in the negative direction, suggesting that L2 verbs with fewer similar counterparts in the L1 lexicon are easier to learn.

**Table 4:** Model for predicting whether learners’ uses of l2 words include the most typical referents (dependent variable: Whether L2 learners map verbs to their typical referents).

Fixed effects	Estimate	Std. error	z value	*p*-value
Intercept	7.03	3.66	1.92	.05
Input frequency	1.43	.88	1.62	.10
Category size	-0.34	.26	-1.31	.19
Category ambiguity	-4.16	2.65	-1.56	.11
L1–L2 semantic similarity	-14.74	5.22	-2.82	.00

The second model predicted the degree to which L2 learners understood the adequate categories of L2 words using the f-measure score with the same fixed and random effects as the first model, adopting a Gaussian linear model. The results of the second model are presented in Table 5. The results showed that input frequency, category size, and category ambiguity had a significant effect. The direction of the factors suggests that L2 learners readily find adequate boundaries of L2 words that are highly frequently input, clearly separated from other neighboring words, have small category sizes, and have fewer similar counterparts.

**Table 5:** Model for predicting whether learners’ uses of L2 words cover the native-like ranges of referents (dependent variable: Degree to which L2 learners understand the adequate categories of L2 words).

Fixed effects	Estimate	Std. error	t value	*p*-value
Intercept	1.88	0.42	4.54	.00
Input frequency	.01	.00	3.20	.00
Category size	-.13	.03	-3.96	.00
Category ambiguity	-1.01	.42	-2.4	.02
L1–L2 semantic similarity	-.96	.56	-1.73	.08

## Discussion

### What factors predict the ease/difficulty of learning L2 verbs in terms of center mapping and boundary delineation?

The current study examined how Mandarin-speaking learners of Japanese learn the semantic boundaries of synonymous words and identified the factors that influence learning the L2 lexical system in terms of center mapping and boundary delineation.

Analysis 1 revealed that L2 learners had great difficulty finding native-like semantic boundaries. The semantic systems of Japanese and Mandarin are so different that learners had difficulity delineationg cutting/breaking events in L2 verbs. In particular, L2 learners tended to use general-purpose verbs such as *kiru* (切る: “cutting an object with an edged tool”) for cutting events and *wakeru* (分ける: “dividing an object into several pieces”) for breaking events, respectively. Previous studies have reported that L2 learners prefer to use general-purpose verbs such as “do” in English to stretch their limited lexical resources to meet communicative demands [1,51]. The present results further revealed that the usage of general-purpose verbs was constrained by perceptually salient distinctions of cutting and breaking; the MDS solutions showed that L2 leaners separated the actions of *wakeru* from those of *kiru* [6]. The results of Analysis 1 also indicated that L2 learners had difficulty organizing the manual breaking events, which Japanese verbs more finely delineate than Mandarin, implying the difficulty for L2 learners to move from simple to complex semantic systems [16,20,28].

To quantitatively examine such semantic factors that may contribute to the ease or difficulty of learning cutting/breaking verbs, model analyses were conducted in Analysis 2. Importantly, the results clearly showed that different sets of factors contribute to the learning process of different aspects of semantic representation, that is, center mapping and boundary delineation. In summary, L1–L2 semantic similarity explained the ease of learning in the center mapping process. Regarding boundary delineation, input frequency, category size, and category ambiguity were significant predictors.

Contrary to our hypothesis, input frequency and category size did not affect the center mapping process, but contributed to the boundary delineation process in the same way as category ambiguity. In light of the category learning process, researchers have documented that learners need to be exposed to sufficient input in specifying the typical meaning of a word (i.e., center mapping) because the overall shape of the statistical distribution of input determines whether the meaning of a word is typical or peripheral [14,15,31,56]. However, the results suggested that this explanation may not apply to learners who learn L2 in an L1-speaking environment. L2 learners in L1-speaking environments generally do not receive sufficient linguistic input to extract the typical exemplars. Instead, they learn the typical meaning of words through classroom instruction or descriptions in dictionaries in their L1. In such a learning enviroment, input frequency and category size have a less significant role than does L1–L2 semantic similarity.

The model analyses revealed that L1–L2 semantic similarity had a significant effect on the center mapping process, which was partially consistent with our prediction. Here, a negative transfer seemed to have occured when semantic categories were partially shared among the lexical domains in L1 and L2 [20,28]. The results were consistent with previous studies explaining that the L1 transfer appears in the early stages of L2 word learning and changes its role according to the stage of lexical development [26]. Specifically, in the initial stages of L2 semantic learning, L2 learners have to learn the typical meaning of L2 words. As the instruction is carried out using the learner’s L1, a negative L1 transfer is likely to occur. However, the situation is different in the boundary delineation process. To understand which word usages are appropriate, L2 learners have to be exposed to a large amount of linguistic input. It is quite difficult to infer the adequate categories of word meaning from top-down instruction; dictionary descriptions or classroom instructions cannot cover every situation in which a word can or cannot be used [7]. Thus, in addition to qualitative aspects of input, quantitative aspects of linguistic input are essential to accomplish the boundary delineation process.

The present study revealed that the important variables in L2 lexical acquisition are quite different depending on which aspect of meaning researchers target. The process of constructing a semantic domain in L2 should be assessed from at least two different perspectives of semantic representation encompassing center mapping and boundary delineation simultaneously. The center mapping process is driven by the factor specific to L2 semantic acquisition, but the process of boundary delineation is established by a domain-general category learning mechanism. Thus, the two processes include factors that are generic to categorical learning at large as well as the domain-specific factors germane to the L2 learning environment.

### Limitations and future directions

One limitation of the current study is the issue of factor selection. In particular, more accurate models could have been constructed if we could determine the input frequency that more accurately reflected the actual frequency L2 learners received in the classroom. To the best of our knowledge, no such data are available, which is why we adopted input frequency from textbooks. Accurate measurements of input frequency would be important for model analyses in the future.

The outcomes of the study raise novel queries for future investigations on L2 vocabulary acquisition. First, it is of the utmost importance to scrutinize other factors that affect the ease or difficulty of learning. One intriguing possibility that should be explored is the effect of perceptual saliency. Gentner and Bowerman [5] proposed the *typological prevalence hypothesis*, which predicted that the perceptually salient distinction reflected in the lexicon of many languages may be readily acquired by children. In the current case, L2 learners could find a distinction that is perceptually clear and cross-linguistically shared [38]. Owing to the current study’s inability to regulate the perceptual features of the events, and previous L2 lexical acquisition research not focusing on this factor, it should be examined in future investigations. Second, to confirm the generalizability of the current results, it is important to examine whether the factors are observed in other semantic domains or languages. It is conceivable that the pattern of the learning process varies from domain to domain in L2 word learning, as research on L1 lexical acquisition has demonstrated that word learning trajectories differ depending on the semantic domain’s characteristics [5,57].

## Supporting information

S1 Table Dominant verbs produced by native speakers and L2 learners. (DOCX)
