# Supplementary material for: Learning semantic categories of L2 verbs: The case of cutting and breaking verbs
Source: PLoS One. 2024 Jan 19;19(1):e0296628. doi: 10.1371/journal.pone.0296628 (PMC10798537; doi:10.1371/journal.pone.0296628)
Supplement: S1 Table — (DOCX) [file pone.0296628.s001.docx]

**S1 Table. Dominant Verbs Produced by Native Speakers and L2 Learners.**

| ID | Japanese | Mandarin | High-proficiency learners | Low-proficiency learners |
| --- | --- | --- | --- | --- |
| 1 | *kiru* (0.9)  (“cutting an object with an edged tool”) | qie1 (0.93)  (cutting with a single-blade instrument) | kiru (0.97) | kiru (0.73) |
| 2 | *kiru* (0.76) | pian4 (0.41)  (slicing with a single-blade instrument) | kiru (0.67) | kiru (0.83) |
| 3 | *kiru* (0.95) | qie1 (0.59) | kiru (0.9) | kiru (0.7) |
| 4 | *tataku* (0.52)  (“chopping an object into fine pieces”) | duo4 (0.97)  (chopping repeatedly) | kiru (0.4) | kiru (0.67) |
| 5 | *kiru* (0.9) | qie1 (1) | kiru (0.9) | kiru (0.9) |
| 6 | *kiru* (0.76) | duo4 (0.52) | kiru (0.63) | kiru (0.6) |
| 7 | *kiru* (0.71) | duo4 (0.69) | kiru (0.8) | kiru (0.53) |
| 8 | *kiru* (1) | qie1 (1) | kiru (0.87) | kiru (0.83) |
| 9 | *karu* (0.81)  (“mowing grass”) | ge1 (0.83)  (cutting with a single-blade instrument, slowly back and forth) | kiru (0.3) | kiru (0.57) |
| 10 | *waru* (0.95)  (“splitting a 2- or 3-dimensional object into several pieces”) | pi1 (0.79)  (hacking with force and into halves) | kiru (0.6) | kiru (0.37) |
| 11 | *kiru* (0.9) | ju4 (0.93)  (cutting with a saw) | kiru (0.67) | kiru (0.57) |
| 12 | *karu* (0.48) | jian3 (0.59)  (cutting with a two-bladed instrument) | karu (0.33) | kiru (0.53) |
| 13 | *kiru* (1) | jian3 (1) | kiru (0.87) | kiru (0.77) |
| 14 | *kiru* (1) | jian3 (1) | kiru (0.63) | kiru (0.7) |
| 15 | *kiru* (0.81) | jian3 (0.72) | kiru (0.57) | kiru (0.53) |
| 16 | *kiru* (1) | jian3 (0.93) | kiru (0.7) | kiru (0.6) |
| 17 | *kiru* (1) | cai2 (0.62)  (cutting a paper-like object into parts with a bladed instrument) | kiru (0.63) | kiru (0.6) |
| 18 | *chigiru* (0.52)  (“tearing an object into small pieces by hand”) | bai1 (0.86)  (bending an object forcefully in a vertical direction by hand) | wakeru (0.63)  (“dividing an object into several pieces”) | wakeru (0.23) |
| 19 | *chigiru* (0.86) | si1 (0.86)  (pulling on a flexible paper-like object with hands) | wakeru (0.63) | kiru (0.2) |
| 20 | *chigiru* (0.81) | si1 (0.55) | wakeru (0.23) | wakeru /kiru |
| 21 | *saku* (0.71)  (“ripping up an object by hand”) | si1 (0.93) | wakeru (0.3) | wakeru (0.17) |
| 22 | *yaburu* (0.48)  (“tearing a thin object by hand”) | si1 (1) | wakeru (0.3) | kiru (0.13) |
| 23 | *saku* (0.43) | si1 (0.86) | wakeru (0.23) | wakeru/kowasu (0.13) |
| 24 | *waru* (0.57)  (“splitting a 2- or 3-dimensional object into several pieces”) | bai1 (0.9) | wakeru (0.73) | wakeru (0.17) |
| 25 | *oru* (0.81)  (“snapping a 1-dimensional object”) | bai1 (0.66) | oru (0.5) | oru (0.5) |
| 26 | *oru* (0.71) | bai1 (1) | oru (0.33) | oru (0.17) |
| 27 | *waru* (0.86) | bai1 (0.97) | wakeru (0.53) | wakeru (0.23) |
| 28 | *waru* (0.9) | bai1 (0.97) | wakeru (0.7) | wakeru (0.27) |
